# Airway fibrin formation cascade in allergic asthma exacerbation: implications for inflammation and remodeling

**DOI:** 10.1186/s12014-022-09351-3

**Published:** 2022-05-19

**Authors:** Yanlong Zhu, Stephane Esnault, Ying Ge, Nizar N. Jarjour, Allan R. Brasier

**Affiliations:** 1 Department of Cell and Regenerative Biology, University of Wisconsin-Madison, Madison, WI 53705 USA; 2 Human Proteomics Program, School of Medicine and Public Health, University of Wisconsin-Madison, Madison, WI 53705 USA; 3 Division of Allergy, Pulmonary and Critical Care Medicine, Department of Medicine, University of Wisconsin-Madison School of Medicine and Public Health (SMPH), Madison, WI 53705 USA; 4 Institute for Clinical and Translational Research (ICTR), University of Wisconsin-Madison, 715 Highland Ave, Madison, WI 53705 USA

**Keywords:** Allergic asthma, Inflammation, Coagulation, Proteomics, Tissue Remodeling

## Abstract

**Background:**

Airway remodeling in patients with asthma, which leads to a decline in pulmonary function, is likely the result of repeated exacerbations often provoked by aeroallergen exposures. Aeroallegen exposure triggers a stereotypic response orchestrated by growth factor cytokines and other protein mediators. This results in a late-phase allergic reaction characterized by vascular permeability, recruitment of activated leukocytes, and activation of structural cells of the airway. The spectrum of protein mediators and their functions are incompletely understood.

**Methods:**

Bronchoalveolar lavage fluid (BALF) samples were obtained from 12 volunteers who exhibited robust eosinophilic recruitment following segmental bronchial provocation with allergen (SBP-Ag). We systematically identified and quantified proteins in BALF using high-performance liquid chromatography–high-resolution mass spectrometry (LC–MS/MS) followed by pathway analysis and correlations with airway physiology.

**Results:**

Pairwise analysis of protein abundance in BALF pre- *vs* post-SBP-Ag revealed that 55 proteins were upregulated and 103 proteins were downregulated. We observed enrichment of groups of proteins mapping to hemostasis/fibrin clot, platelet activation, lipoprotein assembly, neutrophil degranulation proteins, and acute-phase inflammation-airway remodeling pathways. The abundances of F2 and Fibrinogen γ (FGG) correlated with eosinophil numbers, whereas SERPINA3 negatively correlated with change in FeNO. The coagulation proteins F2 and KNG negatively correlated with FN1 an index of airway remodeling. Interestingly, patients with lower FEV_1_ showed distinct allergen-induced patterns of 8 BALF proteins, including MUC1, alarmins (HSPB1), and actin polymerization factors.

**Conclusions:**

Protein abundance of the fibrin formation cascade, platelet activation and remodeling are associated with late-phase leukocyte numbers and markers of remodeling. Patients with lower FEV_1_ have distinct dynamic responses to allergen.

**Supplementary Information:**

The online version contains supplementary material available at 10.1186/s12014-022-09351-3.

## Background

Allergic asthma is a prevalent disease characterized by episodic airway obstruction, hyperresponsiveness, and inflammation [1]. Asthma exacerbations are often provoked by exposure to specific aeroallergens triggering acute inflammation with capillary leak, leukocytic recruitment and remodeling [2]. Although asthma exacerbations are associated with unscheduled health care delivery, increasing appreciation that exacerbations are linked to eventual development of airway remodeling and reduction in lung function over time. Airway remodeling is a complex multicellular response characterized by thickening of the reticular basement membrane, mucus gland hypertrophy, epithelial cell state changes, increased smooth muscle mass, and angiogenesis [3–5]. Through interactions from cytokines and growth factors produced during exacerbation, resident cells undergo phenotypic changes (epithelial-mesenchymal and fibroblast transitions) leading to airway remodeling and decline in pulmonary function in a subgroup of patients [6].

Human studies using segmental bronchoprovocation with allergen (SBP-Ag) have led to an improved understanding of the complex multicellular interactions governing the allergic airway response. Acutely, aeroallergen exposure triggers mediator release from local mast cells, resulting in acute bronchoconstriction [7]. The acute response is then followed by a late-phase reaction involving cellular infiltration (granulocytic and lymphocytic) [8] accompanied by the production of proteins involved in initiation of tissue remodeling [9]. In earlier studies, we have identified increased amounts of multiple cytokines and chemokines [10–12] as well as factors implicated in airway remodeling and fibrin formation, such as YKL40 (CHI3L1), follistatin-like 1, matrix metalloproteinase 9 (MMP9), fibronectin-1 (FN1), thrombin (F2) and factor XIII (F13A1) in bronchoalveolar lavage fluid (BALF) 48 h after SBP-Ag [9, 13–17]. A separate analysis of BALF in 4 asthmatics *vs* 3 normal controls subjected to SBP-Ag identified the presence of chemokines, proteases, acute-phase reactants, and others in the asthmatic BALF [18]. However, this small study was not designed to understand the dynamic changes in asthmatics induced by SBP-Ag.

Because mediators released during the late-phase allergic response likely coordinate the complex multicellular events and characteristic of the late phase inflammation, we hypothesized that SBP-Ag may induce proteins that could yield important insights into the pathophysiology of airway remodeling. To pursue this question, we applied label-free quantitative proteomics providing systematic protein identification and quantification in airway fluids from paired samples before and after SBP-Ag using advanced, high-resolution liquid chromatography—tandem mass spectrometry (LC–MS/MS). Our approach is further supported by the knowledge that direct sampling of bronchoalveolar lavage fluid (BALF) most faithfully reflects the milieu of the late-phase reaction [19]. Here, we analyzed paired BALF samples from 12 volunteers with atopic asthma at rest and after late phase induction. Our study identifies the spectrum of proteins involved in the fibrin formation/coagulation cascade, granulocytic activation, and airway remodeling.

## Methods

### Segmental bronchoprovocation with allergen (SBP-Ag)

The University of Wisconsin-Madison Health Sciences Institutional Review Board (Madison, WI, USA) approved the study, and each participant provided written informed consent. As previously described [20], subjects had mild allergic asthma (aeroallergen skin prick test positive, improvement in forced expiratory volume in 1 s. (FEV_1_) ≥ 12% in response to albuterol, or a 20% fall in FEV_1_ in response to ≤ 8 mg/ml methacholine, prealbuterol FEV_1_ ≥ 70%, and postalbuterol FEV_1_ ≥ 80%) and none of the subjects were using inhaled or oral corticosteroids. Participants underwent whole-lung allergen inhalation challenge (WLAC) to determine AgPD_20_, the allergen provocation dose resulting in a 20% reduction in FEV_1_ within 1 h of challenge. Three allergens were used, including *Dermatophagoides farinae* (house dust mite), GS ragweed mix, or Fel d1 (cat) (all from Greer Labs, Lenoir, NC, USA). One month later, a baseline bronchoscopy with BAL was performed followed by SBP-Ag at a dose of 20% of each subject’s AgPD_20_. Forty-eight hours later, bronchoscopy with BAL was performed in the same challenged segment. The volume of normal saline (0.9% sodium chloride) for BAL was 160 ml and as an average ± SD, 115 ± 15 ml was recovered. Fourteen subjects finished the SBP-Ag protocol and 2 were excluded for lower percentage of EOS in BAL after SBP-Ag (< 15%). BAL cell differentials were determined by counting a total of 1000 cells on two cytospin preparations stained with the Wright-Giemsa-based Hema-3 (ThermoFisher, Pittsburgh, PA, USA). Cell-free BALF were stored at – 80 ºC.

### Materials

Reagents and chemicals were purchased from Sigma-Aldrich, Inc. (St. Louis, MO, USA) unless otherwise noted. HPLC-grade methanol, water and acetonitrile were purchased from Fisher Scientific (Fair Lawn, NJ, USA). Trypsin was purchased from Promega (Madison, WI, USA).

### Sample preparation and workflow

**Fig. 1:**
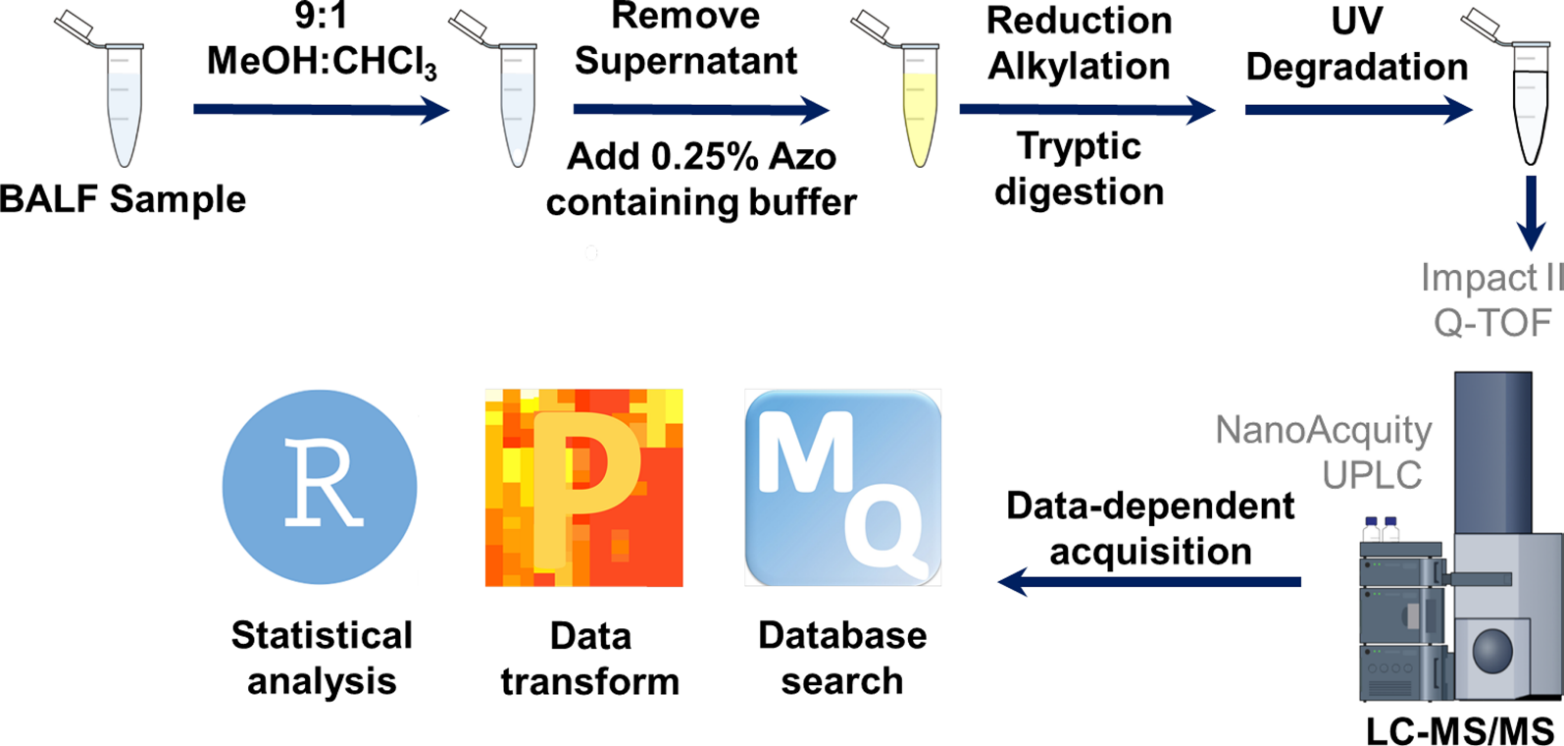
Sample workflow. Schematic view of sample preparation, data acquisition, and data analysis for BALF proteomics

The workflow of sample preparation, data acquisition, and data analysis for BALF proteomics is shown in **Fig. ****1**. BALF samples were thawed on ice. Debris and cells were removed by centrifugation of BALF samples at 13,000 × *g* for 5 min at 4 ºC. 800 µL 9:1 methanol:chloroform was added into 200 µL BALF sample for protein precipitation. The supernatant was removed by centrifugation at 15,000 × *g* for 10 min at 4 ºC. Proteins were re-solubilized in 30 µL buffer containing 0.25% Azo [21, 22], 25 mM ammonium bicarbonate, 5 mM tris(2-carboxyethyl)phosphine (TCEP), 5 mM ethylenediaminetetraacetic acid (EDTA), and 1 × Halt protease/phosphatase inhibitor cocktail. Protein concentration was determined using Bradford protein assay reagent with albumin as a standard and was normalized to 0.5 µg/µL (No normalization for the samples below 0.5 µg/µL). 12.5 µg protein was reduced with 5 mM dithiothreitol (DTT), alkylated with 15 mM iodoacetamide (IAA), and digested with trypsin (1:50) overnight at 37 ºC. The enzymatic activity was quenched with 0.5 µL 10% TFA, and the solution was irradiated with UV light (305 nm) for 5 min to degrade Azo. After centrifugation, supernatant was dried and reconstituted in H_2_O with 0.1% formic acid at 0.5 µg/µL.

### LC–MS/MS analysis of BALF samples

LC–MS/MS experiments were performed using a Bruker Impact II quadrupole time-of-flight (QTOF) mass spectrometer (Bruker Daltonics, Bremen, Germany) coupled to a Waters nanoACQUITY UPLC system (Waters Corporation, Milford, MA, USA). Tryptic peptides were loaded on a Waters ACQUITY UPLC M-Class Symmetry C18 trap column (100 Å, 5 µm, 180 µm × 20 mm) for online desalting, and then were separated using a Waters ACQUITY UPLC Peptide BEH C18 column (130 Å, 1.7 µm, 75 µm × 200 mm). Mobile phase A and B were 0.2% formic acid in H_2_O and 0.2% formic acid in ACN, respectively. Peptides were trapped for 5 min at 6 µL/min flow rate and were separated at 60 ºC using a 300 nL/min flow rate and a stepwise gradient (95% A-0 min, 95%A-5 min, 55% A-60 min, 10% A-75 min, 10% A-82 min, 95% A-85 min, 95% A-95 min). Peptides eluted from the column were infused into the mass spectrometer using a CaptiveSpray nano-electrospray ion source with ACN nanoBooster (Bruker Daltonics, Bremen, Germany). The CaptiveSpray nanoBooster was operated with 0.2 bar ACN-enriched gas, 4 L/min dry gas, 1300 V capillary voltage, and 140 ºC dry temperature. MS spectra were acquired within a mass range of 150–2000 m/z. MS/MS data were acquired in data-dependent acquisition, dynamically selecting top 30 most intense precursor ions from the surveyor scan (MS) for collision-induced dissociation (CID). The same precursor ion was excluded after 2 spectra, and released after 0.5 min.

### Data analysis

LC–MS/MS were searched against the UniProt human database UP000005640 (accessed 22 March 2021) using MaxQuant version 1.6.17.0 [23]. A 1% false discovery rate was used at the peptide and protein level. The minimum peptides length was 7. Carbamidomethylation was selected as a fixed modification and N-terminal acetylation and methionine oxidation were selected as variable modifications. Enzyme specificity was set to trypsin/P and a maximum of 2 missed cleavages were allowed. Match between runs feature was used with a 0.7 min window after retention time alignment to maximize identifications between runs. Classic normalization and a minimum ratio count of 2 were used for label free quantification (LFQ). Data was further processed using Perseus software v. 1.6.5.0 [24]. Potential contaminants and reverse hits were excluded. Log 2 × transformation of LFQ intensity and filtering for valid values were performed. Missing values were imputed using the “replace missing values with normal distribution” function in Perseus using the default parameters (a width of 0.3 and downshift of 1.8).

Significant differences in protein abundance was determined by an empiric Bayes approach using statistical analysis of microarray (SAM) [25]. Statistical significance is adjusted a delta of 0.6 and expressed as a “q-value” adjusted for multiple hypothesis testing. Principal components analysis was in R (v. 3.6). Hierarchical clustering was using log2 transformed LFQ intensity in the pheatmap (version 1.0.12) package in R. Pearson correlation coefficients were calculated and correlograms generated by corrgram package (1.14) in R.

## Results

### Demographics

Volunteers in this study averaged 26 ± 5 years of age and pre-SBP-Ag predicted FEV_1_ was bimodal, with 5 having FEV1 < 87% and 7 with FEV1 of > 95%. Groupwise, the median was 95 ± 16% (Table 1 and Additional file 2: Table S1). After allergen challenge, BAL eosinophil ranged from 17.7 to 80.6% of total BAL cells (eosinophils, neutrophils, lymphocytes and macrophages), and as an average, neutrophil % increased by more than fivefold (Table 1). BAL eosinophil counts rose to 23 ± 21.6 × 10^4^ from 0.01 ± 0.01 × 10^4^ cells/ml, and that of neutrophils rose to 0.9 ± 1 × 10^4^ from 0.02 ± 0.01 cells/ml before challenge. Fraction of exhaled nitric oxide (FeNO) increased to 63.2 ± 16.7 from 41.65 ± 16.28 (Table 1). The proteins in cell-free BALF from pre- and post SBP-Ag were processed and identified by LC–MS/MS (Fig. 1).

**Table 1:** Subjects’ demographics, n = 12 subjects

Subject	Age	SBP-Ag	FEV1 (%)	Total BAL cells (10E4 Cells/mL)	EOS (%)	EOS Change (%) Post–Pre	PMN (%)	PMN Change (%) Post–Pre	LYM (%)	LYM Change (%) Post–Pre	MAC (%)	MAC Change (%) Post–Pre	FeNO
1	23	Pre	75	14.8	1.1		1		3.6		94.3		56
		Post		145.4	74.5	73.4	4.8	3.8	6.9	3.3	13.8	− 80.5	82
2	27	Pre	102	10.4	0.4		0.2		2.5		96.9		19.9
		Post		233.7	73	72.6	1.9	1.7	2	-0.5	23.1	− 73.8	30.1
3	31	Pre	135	7.7	0.5		1		4.8		93.7		41.4
		Post		17.9	51.7	51.2	5	4	7	2.2	36.3	− 57.4	59.8
4	36	Pre	87	8.9	0.4		0.1		4.8		94.7		77.1
		Post		68.3	72	71.6	1.9	1.8	6.8	2	20.2	− 74.5	66.3
5	21	Pre	83	11.1	0.2		0.4		7.2		92.2		49.9
		Post		76.5	77.2	77	1.7	1.3	3	-4.2	18.1	− 74.1	66.8
6	27	Pre	95	7.1	0.4		1.2		8.6		89.8		40.4
		Post		221.7	71.3	70.9	3.2	2	6.6	-2	18.9	− 70.9	57.1
7	27	Pre	71	6.4	1		1.8		14.5		82.7		47.9
		Post		90.7	66.3	65.3	5.4	3.6	8.6	-5.9	19.7	− 63	57
8	19	Pre	103	12.5	0.6		0.9		5.3		93.2		24.5
		Post		193.7	80.6	80	1	0.1	4.8	-0.5	13.6	− 79.6	69.2
9	22	Pre	108	38	0.2		0.7		32.2		66.9		23
		Post		542.6	72.7	72.5	3.6	2.9	14.2	-18	9.5	− 57.4	52.5
10	27	Pre	83	11.3	ND		ND		ND		ND		28.7
		Post		12.2	17.7	ND	9	ND	12.4	ND	60.9	ND	55.6
11	20	Pre	100	8.6	0.6		0.7		8.8		89.9		49.4
		Post		263.5	77.8	77.2	1.4	0.7	12.2	3.4	9.6	− 80.3	99.3
12	33	Pre	99	9.1	ND		ND		ND		ND		ND
		Post		16.3	29.9	ND	2.1	ND	10.6	ND	57.4	ND	ND

*Pre*  pre-segmental bronchoprovocation with an allergen (SBP-Ag), *Post*  48 h post-SBP-Ag, *FEV1 (%)*  forced exhaled volume in 1 s as % predicted obtained prealbutarol and pre-allergen challenge, *EOS* *(%)*  percentage of eosinophils in BAL fluid, *PMN* *(%)*  percentage of neutrophils in BAL fluid, *LYM* *(%)*  percentage of lymphocytes in BAL fluid, *MAC* *(%)* percentage of macrophages in BAL fluid, *FeNO*  fractional exhaled nitric oxide, *ND*  not determined

### Global protein analysis

Proteins whose abundance was significantly changed in the pre *vs* post SBP-Ag BALF were identified by pairwise statistical analysis of microarray (SAM). This pairwise analysis maximized the sample power of the pairwise experimental design and reduced impact of individual proteome variability [25]. Moreover, SAM accommodates the nonparametric distribution of proteins characteristic of proteomics studies [26]. Significant proteins were identified by their wide deviation of the expected vs observed abundance using a high stringency cut-off of ∆ = 0.6 (dashed line in Additional file 1: Fig. S1). Fifty-six proteins were increased by SBP-Ag (red symbols, Additional file 1: Fig. S1); 103 were decreased (green symbols, Additional file 1: Fig. S1). The identification and abundance of the significant proteins for each sample are shown in Additional file 3: Table S2.

**Fig. 2:**
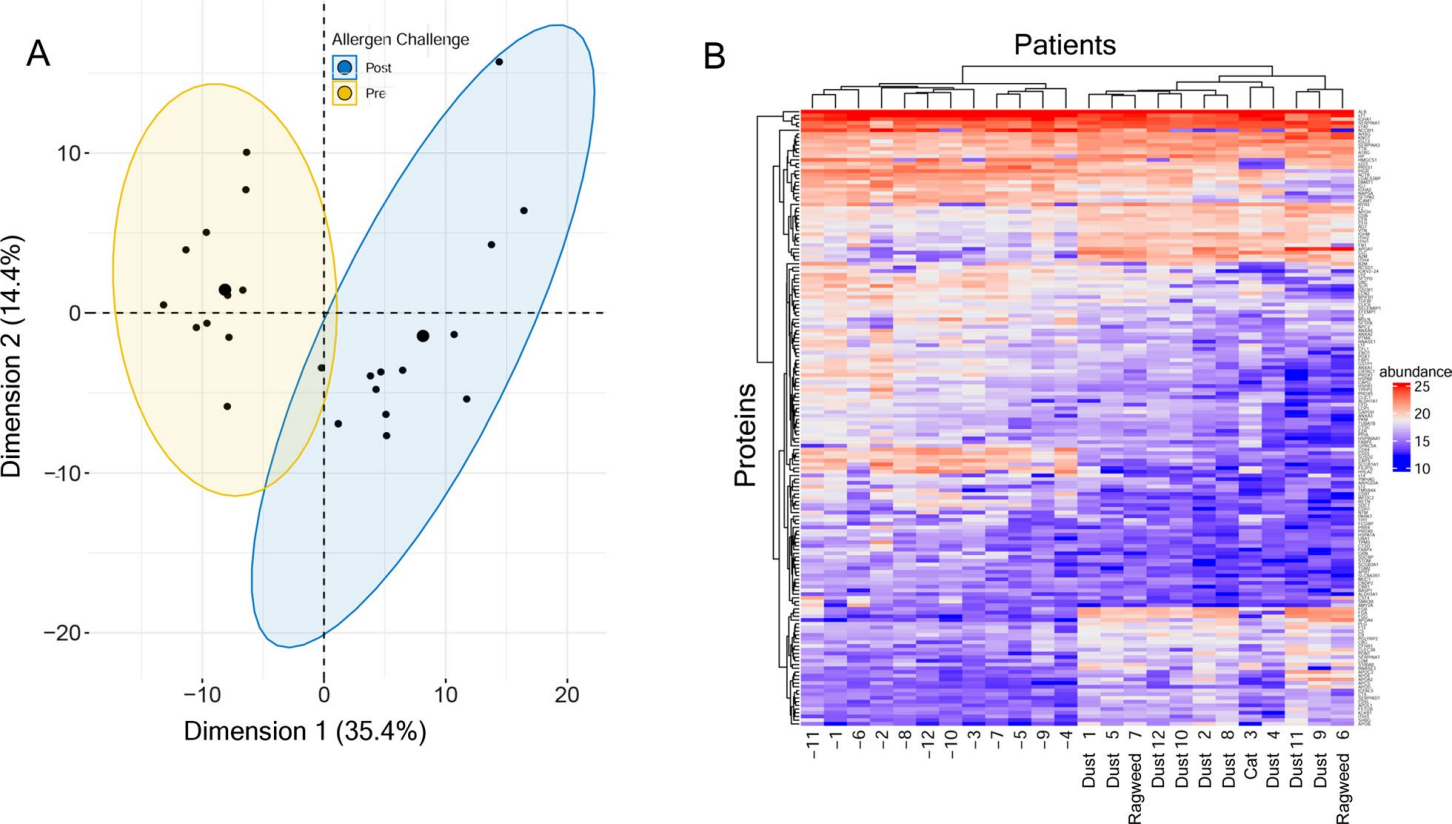
Global expression analysis. **A** Principal Components Analysis (PCA). PCA of 158 significant proteins. Shaded regions indicate PCA for pre- vs post treatment BALF samples. **B** Hierarchical clustering. Shown is hierarchical cluster for 158 significant proteins. Protein abundance was expressed by Log2 transformed LFQ intensity. Note that two major clusters of patients were separated by pre vs post treatment. Patient IDs indicated by “-” are baseline (pre) challenge. The allergens are also listed under the post treatment group

To confirm that the protein patterns distinguished between groups, Principal Components Analysis (PCA) was applied. We observed that the protein abundances in PCA were grouped by SBP-Ag treatment, indicating that protein abundance was informative to late-phase response (Fig. 2A). To further explore the relationship of individual proteins to one another and to SBP-Ag, 2-dimensional hierarchical agglomerative clustering was performed. Here we noted that patients cluster into two clearly distinct groups of baseline BALF (pre-SBP-Ag challenge, indicated by “-” for each individual) and late-phase (post SBP-Ag challenge; Fig. 2B). Similarly, 5 major clusters of proteins emerged. One cluster is a group of proteins high in pre-SBP-Ag challenge that fall after late-phase induction. Several other groups include proteins that were low in basal state and induced by SBP-Ag. These data further indicated that the allergen response was independent of the type of allergen used [27], because the patients with ragweed or cat allergen exposure clustered in the same subgroup as those with dust exposure (Fig. 2B). We concluded from both the PCA and hierarchical clustering results indicate that the protein abundance distinguished the pre- *vs* post allergen challenge samples.

### Pathways enriched in late-phase response

**Fig. 3:**
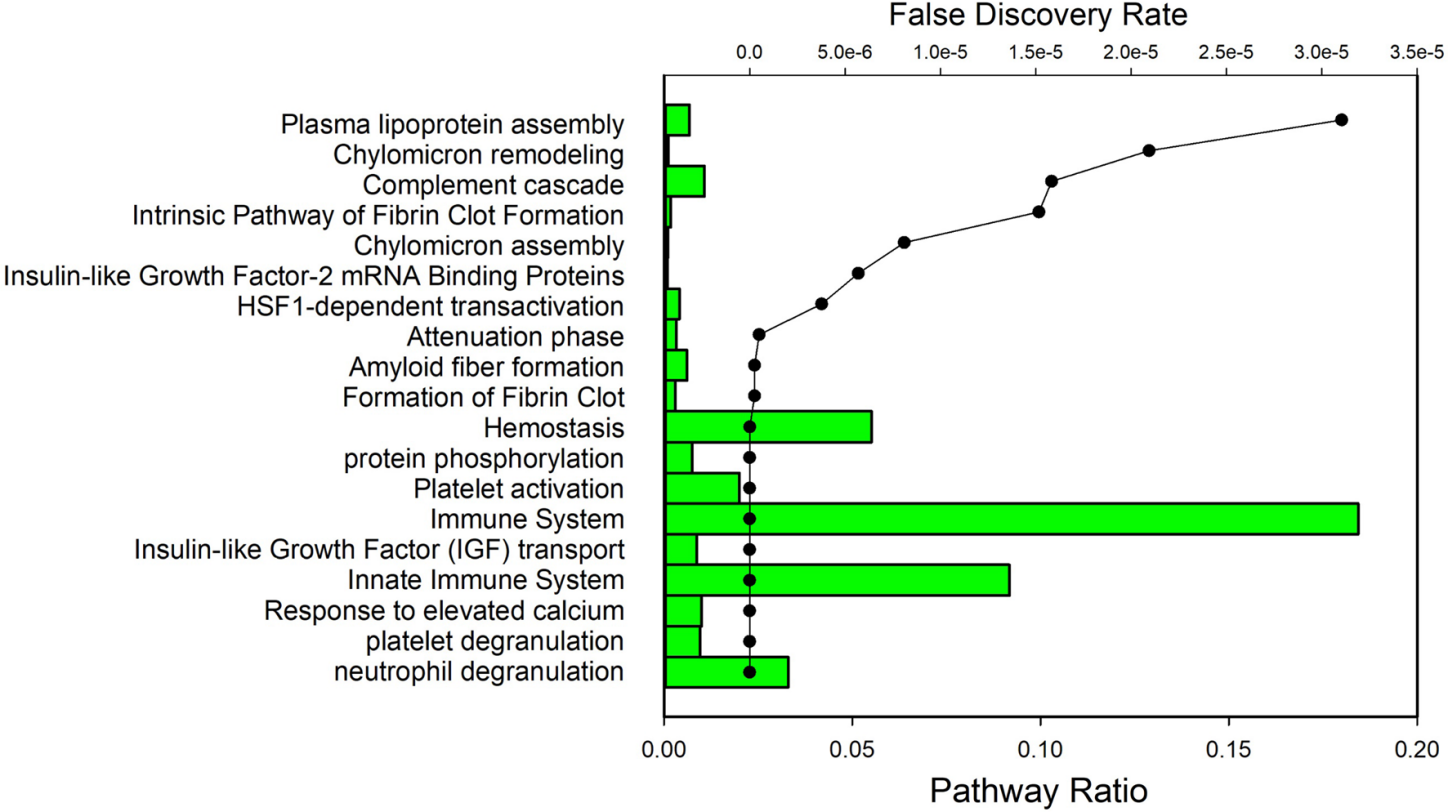
Genome Ontology Analysis. 158 significant proteins were analyzed for pathway enrichment using Reactome.org. The Bars represent pathway ratio, representing the fraction of genes in the BALF data set relative to that of the entire pathway. Significance of enrichment is indicated by false discovery rate (FDR), in scatterplot

**Fig. 4:**
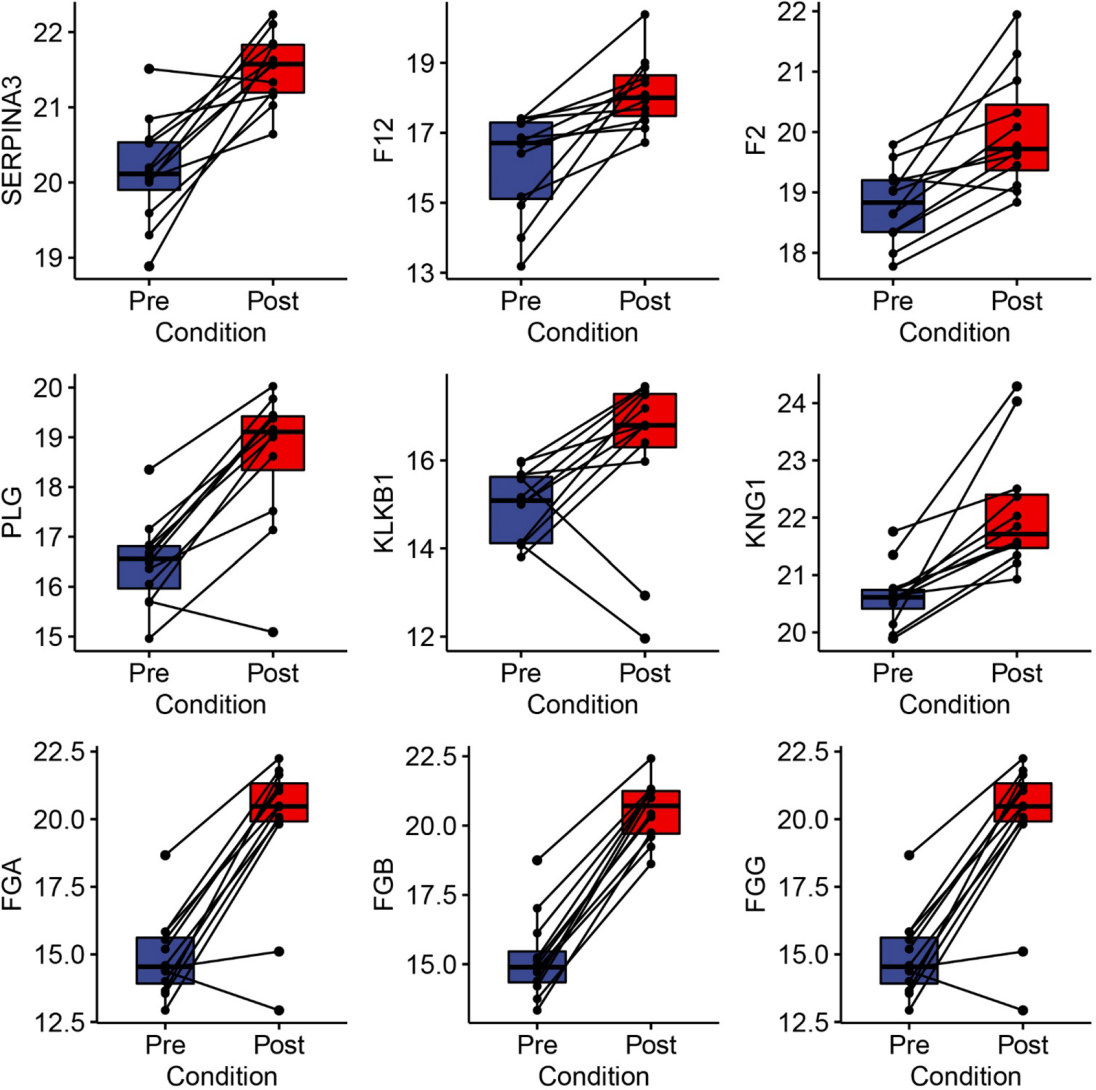
Abundance of BALF proteins involved in fibrin clot formation. Shown are the comparisons of Log2 transformed LFQ intensities of proteins in the fibrin clot formation pathway. Each symbol is a BALF measurement with lines connecting the “pre” with “post” allergen challenge condition for each volunteer. Boxes are 25–75% interquartile range with group median. Abbreviations: SERPINA3, Serpin Family A Member 3, F12, Coagulation Factor XII; F2, Coagulation Factor II (Thrombin), A2M, alpha 2 macroglobulin; KLKB1, Kallikrein B1; KNG, kinonogen; FG, fibrinogen

To understand biological pathways involved in the response to allergen challenge, the 158 significant proteins were analyzed for pathway enrichment relative to the human proteome (Fig. 3). The top ten identified included “neutrophil degranulation” [false discovery rate (FDR) < 2.6 E−14], “platelet degranulation” (FDR < 2.6 E−14), “innate activation” (FDR < 2.6 E−14) and “immune system” (FDR = 1.42 E−11). Multiple entries were identified for fibrin clot formation including, “hemostasis” (FDR = 1.09 E−09), “formation of fibrin clot” (FDR = 2.53 E-07) and “intrinsic pathway of fibrin clot formation” (FDR = 1.52 E−05) (Fig. 3). Similarly, multiple entries were identified for lipoprotein particle formation, including “chylomicron assembly” (FDR = 8.11 E−06), “plasma lipoprotein assembly” (FDR = 3.1 E−05) and “chylomicron remodeling” (FDR = 2.9 E−05) (Fig. 4). Because granulocyte degranulation and innate/immune system activation have been well described, we focused on proteins contributing to less well-studied pathways in asthma.

### Hemostasis/fibrin clot formation

Dysregulation of coagulation and impaired fibrinolytic pathways have been previously observed in acute lung injury, including ARDS [28]. We therefore focused on examining the over 12 index proteins constituting the fibrin clot/hemostasis pathway, all of those abundance was substantially increased as a result of SBP-Ag. Specifically, in pre SBP-Ag BALF, median SERPINA3 abundance was 20.11 ± 0.63 [Log2 LFQ median ± interquartile ratio (IQR)] *vs* post SBP-Ag abundance of 21.6 ± 0.63 IQR (Fig. 4). Similarly, Coagulation Factor XII (F12) changed from 16.7 ± 2.2 to 18.0 ± 1.2 after SBP-Ag (Fig. 4). Coagulation Factor II (F2, Thrombin) changed from 18.8 ± 0.9 to 19.7 ± 1.1 after SBP-Ag (Fig. 4). Plasminogen (PLG) changed from 16.6 ± 0.9 to 18.11 ± 1.1 after SBP-Ag (Fig. 4). Kallikrein B1 (KLKB1) increased from 15.1 ± 1.5 to 16.8 ± 1.2 (Fig. 4). Similar findings were observed for kininogen (KNG) and multiple fibrinogen subunits -alpha (FGA), -beta (FGB) and gamma (FGG), Fig. 4. We noted that FGB showed a uniform induction in all patients, with FBA and FBG showing a more heterogeneous induction in 10 of the 12 patients.

### Correlations with cellular inflammation

**Fig. 5:**
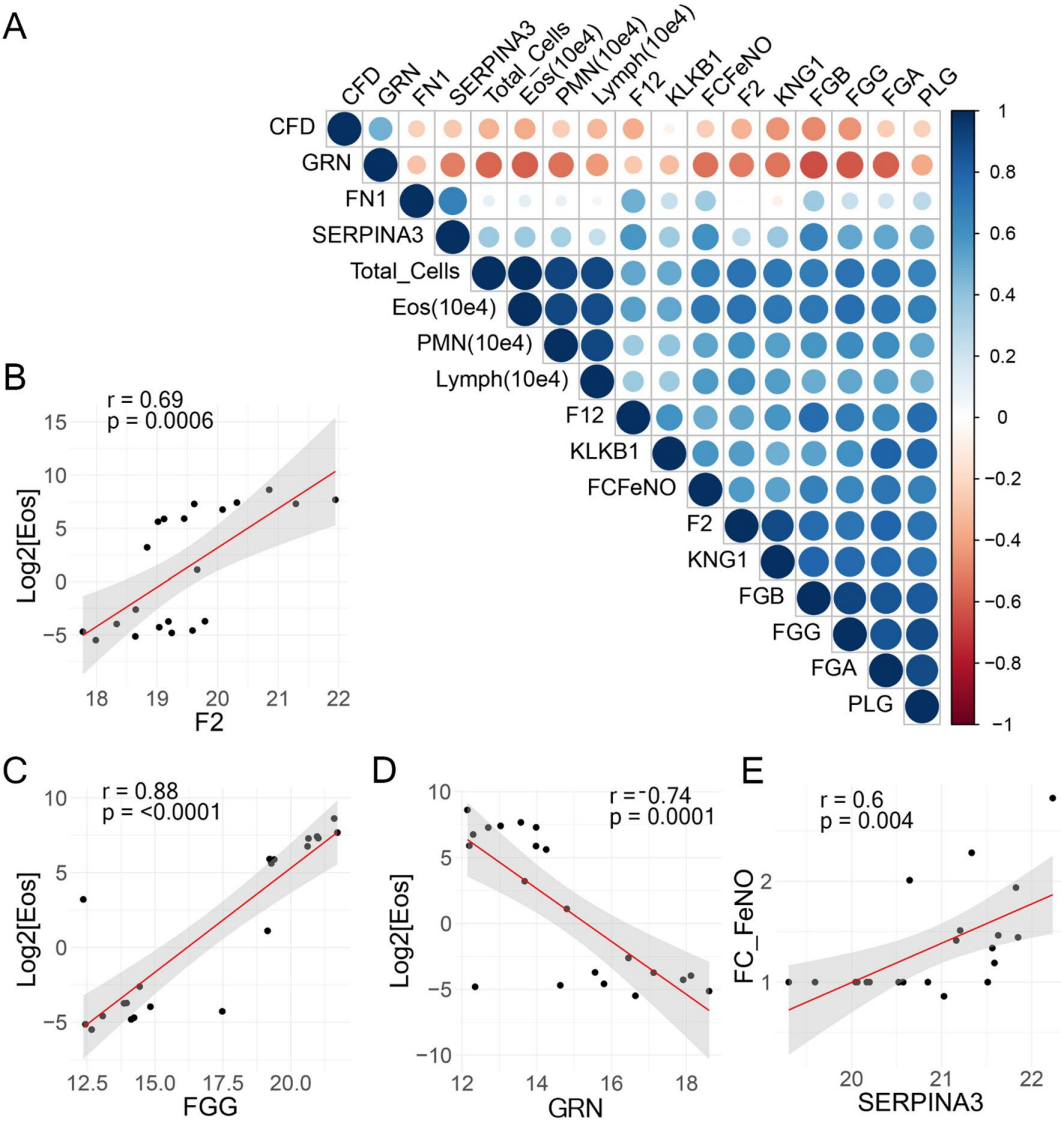
Correlation of fibrin clot pathway with phenotypic features. BALF protein abundance was subjected to correlation analysis with phenotypic measurements of airway physiology and BAL immune cell counts (Eos, eosinophil; PMN, neutrophil; Lymph, lymphocyte). **A** Correlation matrix of BALF protein abundance and phenotypic characteristics. Each sample pre and post was included in the correlation. For each pairwise comparison, the Pearson correlation coefficient is indicated by the color scale (at right). **B**–**G** Correlation plots of highly significant associations. **B** Change in Log2 transformed absolute count of eosinophils (X 10^4^/ml; Log2[Eos]) vs F2 abundance. Scatterplot and linear regression (in red) shown. Confidence interval of the regression is indicated by shading. Pearson correlation and statistical significance are indicated by r, and p, respectively. **C** Log2[Eos] *vs* FGG. **D** Log2[Eos] vs GRN. **E** FCFeNO vs SERPINA3

To better understand the complex relationships between the fibrin formation/hemostatic proteins with measures of airway function and immune cell accumulation, a systematic correlation analysis was conducted. Here, the Pearson correlations of the abundance of the fibrin formation/hemostatic proteins of all samples (pre and post SBP-Ag) were calculated for total cell counts in the same sample and the fold change in FeNO (FCFeNO). A correlogram of all the Pearson correlation for the comparisons is shown in Fig. 5. Total cell counts were highly positively correlated with eosinophil counts, neutrophil counts and lymphocyte counts (indicated as blue in Fig. 5A); and positive correlations between eosinophil number and FCFeNO were seen, increasing the confidence of the correlation study.

Examining the coagulation pathway proteins, F2, FGG, KNG were strongly correlated with eosinophil numbers in all of the pre- and post SBP-Ag samples. A significant and strikingly strong positive correlation was observed between the change in fibrinogen (FG)-B and G isoform abundance with neutrophil numbers. These correlations were subjected to linear regression after regularization of the eosinophil count by Log_2_ transformation. By contrast, granulin precursor (GRN) showed a strong negative correlation with total cells, eosinophils and neutrophils as well as FG isoforms. The Pearson correlation coefficient (r) of F2 with eosinophil number was 0.69 (p = 0.0006, Fig. 5B). Similarly, FGG exhibited an r = 0.88 (p < 0.0001; Fig. 5C). In contrast, GRN exhibited a strong, negative correlation with eosinophil number, (r = − 0.74, p = 0.0001; Fig. 5D). SERPINA3 showed a positive correlation with FCFeNO (r = 0.6, p = 0.004; Fig. 5E).

### Lipoprotein particle formation

**Fig. 6:**
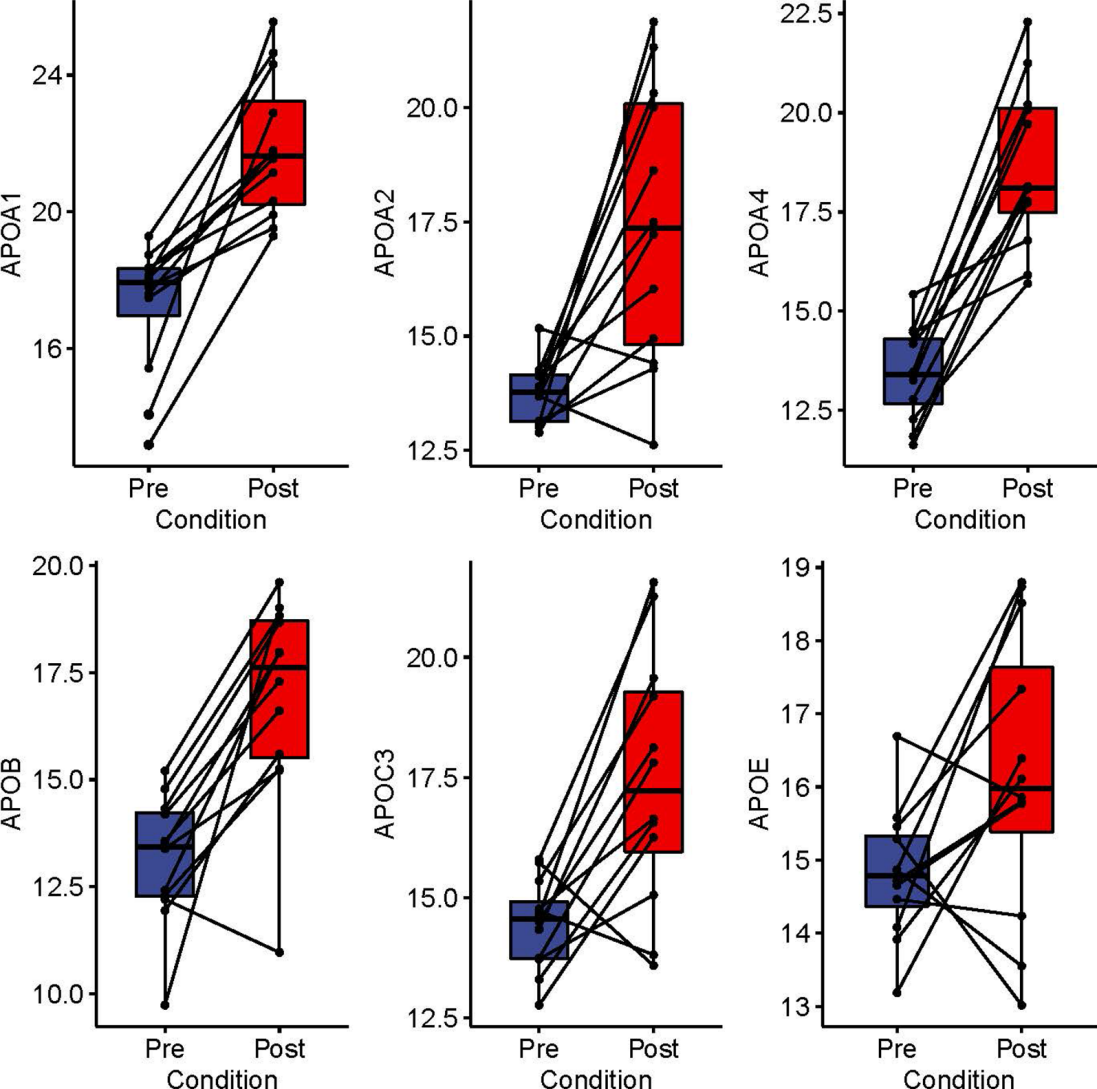
Induction of the apoliprotein assembly pathway. The comparisons of Log2 transformed LFQ intensities of proteins in lipoprotein assembly. Plot as in Fig. 4. Abbreviations: Apo, apolipoprotein

Understanding that Apolipoprotein E (ApoE) has been shown to be a negative regulator of airway hyperreactivity and globlet cell hypertrophy in response to house dust mite exposure [29], we focused on 7 apolipoprotein subunits whose abundance we identified to be changed by SBP-Ag (Additional file 3: Table S2). Here, ApoA1 increased from 17.4 ± 1.4 to 21.6 ± 3.0 (Log2 LFQ median and IQR) after SBP-Ag (Fig. 6). Likewise, ApoA2 increased from 13.8 ± 1.0 to 17.4 ± 5.3; ApoA4 increased from 13.4 ± 1.6 to 18.2 ± 2.6; ApoB increased from 13.4 ± 2 to 17.7 ± 3.2; ApoC3 increased from 14.6 ± 1.2 to 17.2 ± 3.3; and ApoE increased from 14.8 ± 1.0 to 16 ± 2.2 (Fig. 6). We noted from examination of the pairwise plots that induction of ApoA1 and ApoA4 was seen in all late-phase BALFs, whereas ApoC3 and ApoE showed greater variability. Interestingly to us, no significant correlations were observed with FeNO measurement or inflammatory cell counts (not shown).

### Fibrin pathway proteins are inversely related to airway remodeling proteins

**Fig. 7:**
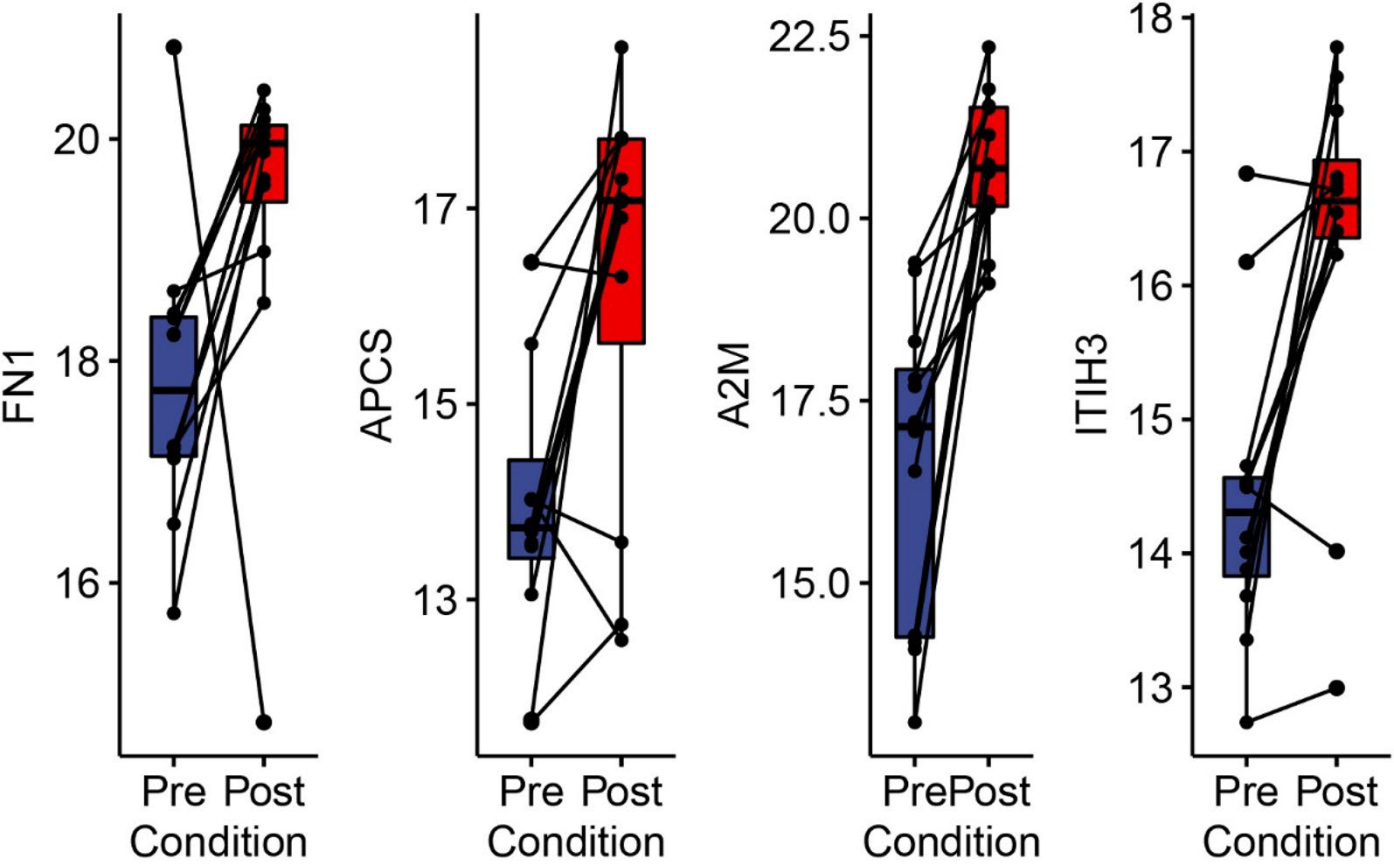
Induction of remodeling proteins. The abundance of empirically identified remodeling proteins representing several genome ontology pathways are plotted using the pairwise representation. FN1, fibronectin; APCS, Amyloid P Component, Serum; A2M, alpha 2 macroglobulin; ITIH3, Inter-Alpha-Trypsin Inhibitor Heavy Chain 3

Systematic proteomic study of BALF in a rodent model of inflammation-induced airway remodeling identified ECM and acute phase proteins that reversed with anti-remodeling treatment [30] and were validated in BALF of patients with severe asthma [5]. The expression of fibronectin (FN1); Amyloid P Component of serum (APCS), alpha2 macroglobulin (A2M) and Inter-Alpha-Trypsin Inhibitor Heavy Chain 3 (ITIH3) were strikingly enriched by SBP-Ag. Specifically, FN1 increased from 17.7 ± 1.3 to 20 ± 0.7 after SBP-Ag (Fig. 7). A2M increased from 17.1 ± 3.7 to 20.7 ± 1.4 after SBP-Ag (Fig. 7); APCS increased from 13.7 ± 1.0 to 17.1 ± 2.1 after SBP-Ag (Fig. 7); and ITIH3 increased from 14.3 ± 0.7 to 16.7 ± 0.6 after SBP-Ag (Fig. 7).

**Fig. 8:**
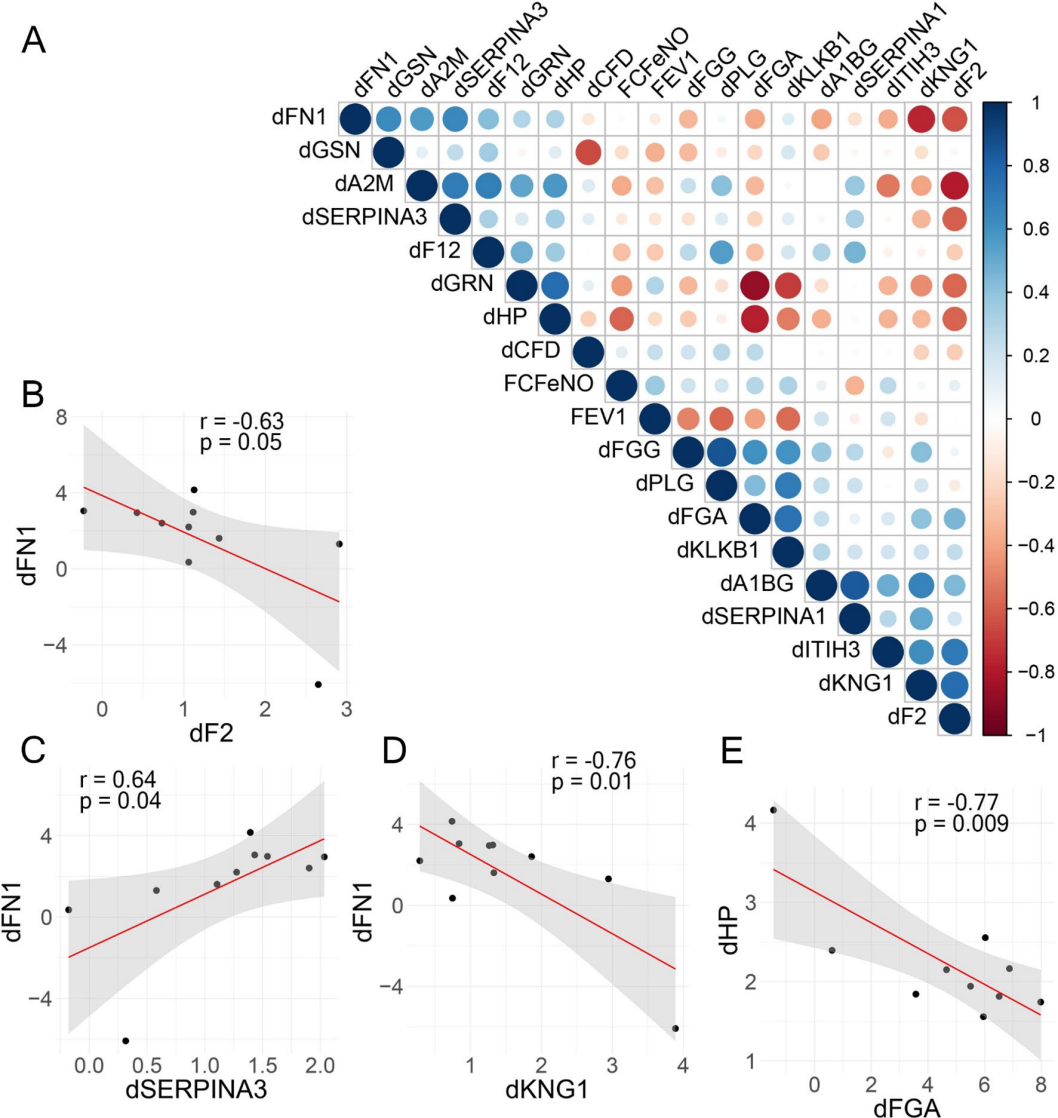
Correlation of remodeling protein expression with phenotypic features. **A** Correlation matrix of changes in remodeling protein abundance (post vs pre, indicated by “d” = ∆) and changes in coagulation protein. For each pairwise comparison, the correlation coefficient is represented (scale at right). Note the position association of dFN1 and dSERPINA3, and negative association with dF2 and dKNG1. dHP is inversely related to dFGA. **B**–**E** correlation plots of highly significant associations. **B** dFN1 vs dF2. **C** dFN1 vs dSERPINA3. **D** dFN1 vs dKNG1. **E** dHP vs dFGA. Regression lines and confidence intervals as in Fig. 5

To establish whether abundance of fibrin pathway proteins was related to indices of airway remodeling, we examined in-depth the relationship with changes in abundance of the fibrin forming proteins and changes in remodeling factors (indicated by “d” in Fig. 8). Of these, FN1 is well established to indicate the presence of airway remodeling. We noted that changes in abundance of FN1 produced by SBP-Ag was positively correlated with changes in SERPINA3 and negatively correlated with SBP-Ag induced changes in KNG1 and F2 (Fig. 8A). Linear regression curves were calculated. Changes in F2 exhibited a negative correlation with FN1 (r = − 0.63, p = 0.05; Fig. 8B), whereas changes in SERPINA3 exhibited a positive correlation with FN1 (r = 0.64, p = 0.04; Fig. 8C). KNG1 was negatively correlated with FN1 (r = − 0.75, p = 0.01; Fig. 8D). Finally, changes in FGA were negatively correlated with haptoglobin (HP), which is an pleiotrophic acute-phase protein [31, 32] involved in inflammation, antioxidant protection and associated with fibroblast differentiation in the airways [33] (r = − 0.77, p = 0.009; Fig. 8E).

### Distinct patterns in patients with low FEV1

**Fig. 9:**
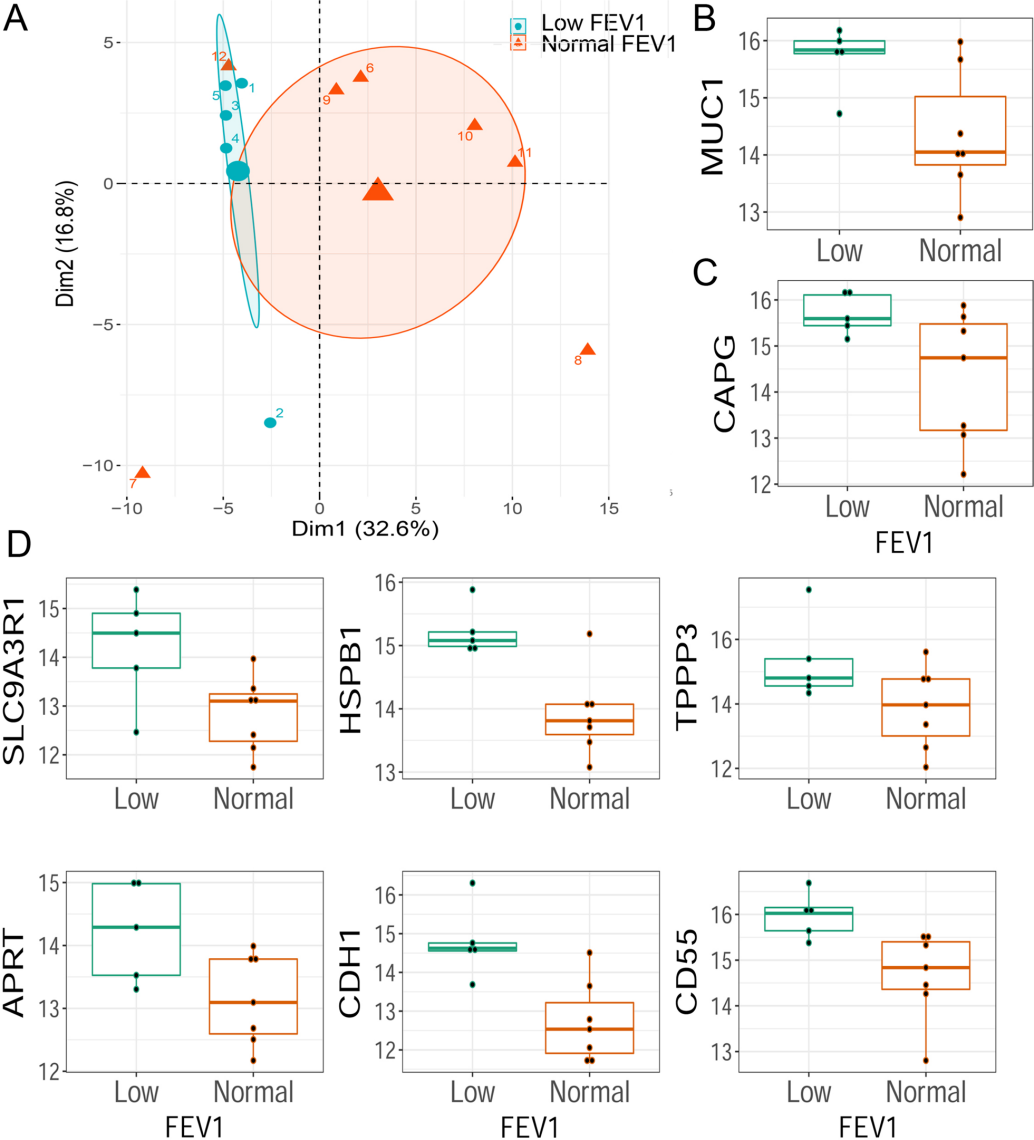
Distinct patterns of allergen-induced BALF proteins by resting FEV_1_. **A** PCA analysis of post BALF samples, labeled by volunteer number. Volunteers with low FEV_1_ (< 87%) are tightly grouped by differences in the first principal component. **B**, **C** Box plots of the significant proteins differentiating low vs normal FEV_1_. **B** Levels of MUC1 were 15.8 ± 0.2 in Low FEV_1_ group *vs* 14.0 ± 0.2 in the normal FEV_1_ group (p = 0.05). **C** Levels of CAPG were 15.6 ± 0.7 in the Low FEV_1_ group *vs* 14.7 ± 2.3 in the normal FEV_1_ group (p = 0.03). **D** Levels of SLC9A3R1 were 14.5 ± 1.1 *vs* 13.1 ± 1.1 (p = 0.04). Levels of HSPB1 were 15.1 ± 0.2 *vs* 13.8 ± 0.5 (p = 0.005). Levels of TTP3 were 14.8 ± 0.8 *vs* 14 ± 1.8 (p = 0.04). Levels of APRT were 14.3 ± 1.5 *vs* 13.1 ± 1.2 (p = 0.05). Levels of CDH1 were 14.6 ± 0.2 *vs* 12.5 ± 1.3 (p = 0.01). Levels of CD55 were 16.0 ± 0.5 *vs* 14.8 ± 1 (p = 0.04). Abbreviations: MUC1, mucin 1; CD55, CD55 molecule/decay accelerating factor; TPPP3, Tubulin Polymerization Promoting Protein Family Member 3; CAPG, Capping Actin Protein, Gelsolin Like; HSPB1, Heat Shock Protein Family B (Small) Member 1; SLC9A3R1, solute carrier SLC9A3 Regulator 1; APRT, Adenine Phosphoribosyltransferase; CDH1, Cadherin 1

The clinical characteristics of subjects enrolled in this study were bimodal with 5 having reduced FEV_1_ (< 87% predicted) and 7 with preserved FEV_1_ (Table 1). In addition, we noted that volunteers with low FEV_1_ (e.g., subject #s 1, 5, 7) clustered together in the hierarchical clustering on the basis of post-SBP-Ag protein abundance (Fig. 2). We therefore further explored whether this group had distinct patterns of protein expression post SBP-Ag. PCA of the post SBP-Ag protein samples showed a tight clustering of subjects numbered 1, 5, 3, 4 and 2 whose separation from normal is based on the first PCA Dimension (Fig. 9A). We therefore examined the proteins that contributed to this difference, revealing mucin 1 (MUC1); CD55 molecule/decay accelerating factor, Tubulin Polymerization Promoting Protein Family Member 3 (TPPP3); Capping Actin Protein, Gelsolin Like (CAPG), Heat Shock Protein Family B (Small) Member 1 (HSPB1); SLC9A3 Regulator 1 (SLC9A3R1), Adenine Phosphoribosyltransferase (APRT) and Cadherin 1 (CDH1) were induced to higher levels in the post SBP-Ag samples (Figs. 9B–E).

## Discussion

Asthma is heterogeneous in its etiology, onset and exacerbating features [34]. In allergic asthma, episodic clinical decompensations are often provoked by aeroallergen exposures that trigger acute inflammation with capillary leak, leukocyte recruitment and potential contribution to eventual airway remodeling. Through interactions from cytokines, resident cells undergoing phenotypic changes (epithelial-mesenchymal and fibroblast transitions) and leukocyte recruitment, repeated episodes of acute exacerbations are associated with structural remodeling and decline in pulmonary function in a subgroup of patients [6]. To further understand the complex coordinated activities of late-phase allergic response, we systematically analyzed the protein expression patterns of the allergic late-phase response in a human challenge model. In addition to identifying known pathways of granulocyte degranulation, innate and adaptive immune responses, we provide evidence for fibrin clot formation, lipoprotein assembly and structural remodeling factors.

Earlier we identified the upregulation of factor XIII in allergic asthma and demonstrated that its abundance correlates with measures of pulmonary function (FEV1/FVC and reversibility of obstruction) and with markers of Th2 activity (IL13 and eosinophil influx) [17]. Factor XIII is a transglutaminase that stabilizes fibrin clot and extracellular matrix components, FN1, thrombospondin and others [35]. In addition, studies have shown the upregulation of tissue factor by IL-13 in Th2-type asthma [36] and enhanced thrombin formation [37], contributing to a pro-coagulant environment. Several human studies have reported presence of an excessive pro-coagulation activity in the airways with plasma exudation as a potential source of the pro-coagulation proteins, particularly in severe asthma [36, 38–40]. Also, complementary studies in small animal models indicate vascular leak in inflammation is produced by cell state changes of capillary pericytes [30]. Our findings in this current study substantially extends the spectrum of proteins in the fibrin formation/coagulation system present in the late-phase BALF including Factor X, XII, FGA, FGB and FGG. These findings are significant because fibrin plugs are found in casts in children with plastic bronchitis [41], and play important roles in the response acute lung injury [28]. Additionally, in support of the strong correlation between FGA, FGB and neutrophil numbers, it has been recently reported that fibrin regulates neutrophilic inflammation in oral mucosal tissues [42]. In that study, insufficient clearance of extravascular fibrin deposits engages neutrophils resulting in the production of oxidative tissue damage. Our findings of fibrinogen in the BALF from aeroallergen challenge suggests that fibrin may also regulate neutrophil effector function in asthma exacerbations. We note that fibrin clots are a component of airway mucus plugging in fatal *status asthmaticus* and is associated with asthma exacerbation and airway obstruction [43, 44]. Our study was not designed to identify the sources of these products but may be due to local synthesis or selective vascular leak.

A previous study employed LC-MS proteomics of BALF in a small group of asthmatics (n = 4) *vs* normal controls exposed to SBP-Ag [18]. We note that this study observed increased complement factors, acute-phase reactants (serum amyloid A, orosomucoid), and apolipoproteins. However, this study is limited in several ways: (1). the protein processing depleted high abundance proteins, including SERPIN and HP, before differential analysis. (2). the study design compared SBP-Ag of asthmatics vs normals, precluding the understanding of what proteins are present in controlled asthma vs those induced by SBP. (3). only 2 of the asthmatics had a robust late-phase response. Consequently, the pairwise design of study enables the understanding of dynamic changes in proteins induced by SBP-Ag in asthmatics and has substantially greater power.

Our unbiased analysis identified substantial enrichment of 7 Apo isoforms in the late-phase BALF. In addition to its role in lipoprotein metabolism, ApoE is a complex multifunctional protein that both promotes and inhibits airway inflammation. For example, ApoE-deficient mice show exaggerated inflammation and airway hyperreactivity in response to house dust mite exposure [29]. In this model, administration of ApoE mimetic peptides reduces goblet cell hyperplasia via an LDLR receptor pathway. Other studies have implicated ApoE as a danger signal, activating NLRP3 inflammasome and IL-1β secretion in pulmonary macrophages in a dose-dependent mechanism in allergic asthmatics [45]. Our analysis raises the possibility that other apolipoprotein isoforms are induced by aeroallergen exposure whose functions may need to be studied in more depth.

Deposition of large amount of extracellular matrix (ECM) is a characteristic of asthmatic airways [46–48]. In a model of inflammation-induced airway remodeling, we earlier conducted a systematic pharmacoproteomics analysis to identify soluble proteins involved in extracellular matrix remodeling. In that previous study, we provided evidence that repeated episodes of innate inflammation triggered by TLR3 ligation resulted in mesenchymal transition mediated by the bromodomain containing BRD4 protein [30]. The synthesis of BRD4-dependent BALF proteins were validated in humans with severe asthma [5]. Here we demonstrate that these remodeling factors are induced by acute allergen challenge, providing a potential linkage between repetitive inflammation and remodeling. Repeated episodes of acute exacerbations are associated with structural remodeling and decline in pulmonary function in a subgroup of patients [6]. The current study also confirm our previous data reporting increase production of FN1 after SBP-Ag [16]. Our correlation studies indicate a strong negative relationship between thrombin, fibrin and FN1, indicating that these proteins may have distinct sources of cellular production, or the consumption of fibrin producing proteins is related to severity of inflammation and tissue injury.

A surprising finding is that patients with lower FEV_1_ expressed 8 proteins at greater levels in the BALF than do patients with preserved FEV_1_. This protein group includes proteins involved in cell stress response (HSPB1), mucosal protection and allergy (MUC1), complement activation (CD55) and actin polymerization (CAPG, APRT, TPPP3). We also note the substantial induction of CDH1, an epithelial derived adhesion factor that plays an important role in airway remodeling and lung function in asthma. For example, it is well established that CDH1 gene polymorphisms are associated with airway remodeling, inflammation and lung function decline in individuals with asthma [49]. These effects are thought to be through disruption of epithelial barrier function and may be restored by inhaled corticosteroids. Importantly, corticosteroid use was an exclusion criteria for entry into this study. The impact of CDH1 gene polymorphisms, reduced FEV_1_ and enhanced CDH1 secretion into the BALF may be of interest to examine in a larger cohort. Our findings suggest that SBP-Ag may induced enhanced epithelial disruption in patients with lower pulmonary functions.

The number of subjects is a limitation for this study. However, since 10 to 20 subjects have been typically enough for correlative analyses in previous studies [14, 17, 20, 50], and due to the difficulty to recruit more subjects during COVID-19 pandemic, we included 12 subjects in the present study. Nevertheless, this preliminary study allows us to assess the feasibility and power to achieve broader analyses of the findings in a larger study. Power calculations evaluate that we will need 20 subjects and 50 subjects for correlations of 0.6 and 0.4, respectively, to reach statistical significance (2-sided p-value < 0.05) with 80% power.

## Conclusion

Our study shows the induction of fibrin formation/coagulation, extracellular matrix protein and apolipoprotein pathways in response to SBP-Ag challenge in allergic asthmatics. The study also identifies new candidates that likely participate in the initiation of airway obstruction (airway plugging and remodeling) and potentially loss of lung function following repeated airway inflammatory responses in asthma.

## Supplementary Information

**Additional file 1: Figure S1.** Statistical Analysis of Microarray. SAM plot of expected vs observed abundance of proteins were plotted. Dashed lines are cut-off for ∆=0.6, corresponding to Q<0.5. Red dots are proteins upregulated by SBP-Ag; green are downregulated.

**Additional file 2:** **Table S1.** Subjects’ demographics with the calculations of averages and their standard deviation (SD). The counts of different cells were calculated in 200 μL BALF.

**Additional file 3:** **Table S2.** The identification and abundance of the significant proteins for each sample.

## Data Availability

The datasets used and/or analysed during the current study are available from the corresponding author on reasonable request.

## Abbreviations

A2M: Alpha 2 macroglobulin
APCS: Amyloid P Component, Serum
Apo: Apolipoprotein
APRT: Adenine Phosphoribosyltransferase
Azo: 4-Hexylphenylazosulfonate
BALF: Bronchoalveolar lavage fluid
CAPG: Capping Actin Protein, Gelsolin Like
CD55: CD55 molecule/decay accelerating factor
CDH1: Cadherin 1
DTT: Dithiothreitol
EDTA: Ethylenediaminetetraacetic acid
EOS: Eosinophils
F12: Coagulation Factor XII
F2: Coagulation Factor II
FC: Fold change
FeNO: Fractional exhaled nitric oxide
FEV_1_: Forced exhaled volume in 1 s
FG: Fibrinogen
FN1: Fibronectin-1
HP: Haptoglobin
HSPB1: Heat Shock Protein Family B (Small) Member 1
IAA: Iodoacetamide
ITIH3: Inter-Alpha-Trypsin Inhibitor Heavy Chain 3
KLKB1: Kallikrein B1
KNG: Kinonogen
LFQ: Label free quantification
LYM: Lymphocytes
MS: Mass spectrometry
MUC1: Mucin 1
PMN: Neutrophils
SAM: Statistical analysis of microarray
SBP-Ag: Segmental bronchoprovocation with an allergen
SERPINA3: Serpin Family A Member 3
SLC9A3R1: Solute carrier SLC9A3 Regulator 1
TCEP: Tris(2-carboxyethyl)phosphine
TPPP3: Tubulin Polymerization Promoting Protein Family Member 3
