# Supplementary material for: Airway fibrin formation cascade in allergic asthma exacerbation: implications for inflammation and remodeling
Source: Clin Proteomics. 2022 May 19;19:15. doi: 10.1186/s12014-022-09351-3 (PMC9117591; doi:10.1186/s12014-022-09351-3)

Supporting Information
Airway Fibrin Formation Cascade in Allergic Asthma Exacerbation: Implications for Inflammation and Remodeling

Yanlong Zhu, PhD[1,2*]

Stephane Esnault, PhD[3*]

Ying Ge, PhD[1,2]

Nizar N Jarjour, MD[3€]

Allan R. Brasier, MD[4€]

[1]Department of Cell and Regenerative Biology, University of Wisconsin-Madison, Madison, Wisconsin 53705, USA

[2]Human Proteomics Program, School of Medicine and Public Health, University of Wisconsin-Madison, Madison, Wisconsin 53705, USA

[3]Division of Allergy, Pulmonary and Critical Care Medicine, Department of Medicine, University of Wisconsin-Madison School of Medicine and Public Health (SMPH), Madison, WI, 53705, USA;

[4]Institute for Clinical and Translational Research (ICTR), University of Wisconsin-Madison, Madison, WI, 53705, USA

* co first authors

€ co senior authors

Supporting Information:

Figure 1. Statistical Analysis of Microarray. SAM plot of expected vs observed abundance of proteins were plotted. Dashed lines are cut-off for Δ=0.6, corresponding to Q<0.5. Red dots are proteins upregulated by SBP-Ag; green are downregulated.

Table 1. Subjects' demographics with the calculations of averages and their standard deviation (SD). The counts of different cells were calculated in 200 µL BALF.

Table 2. The identification and abundance of the significant proteins for each sample.

Figure 1.

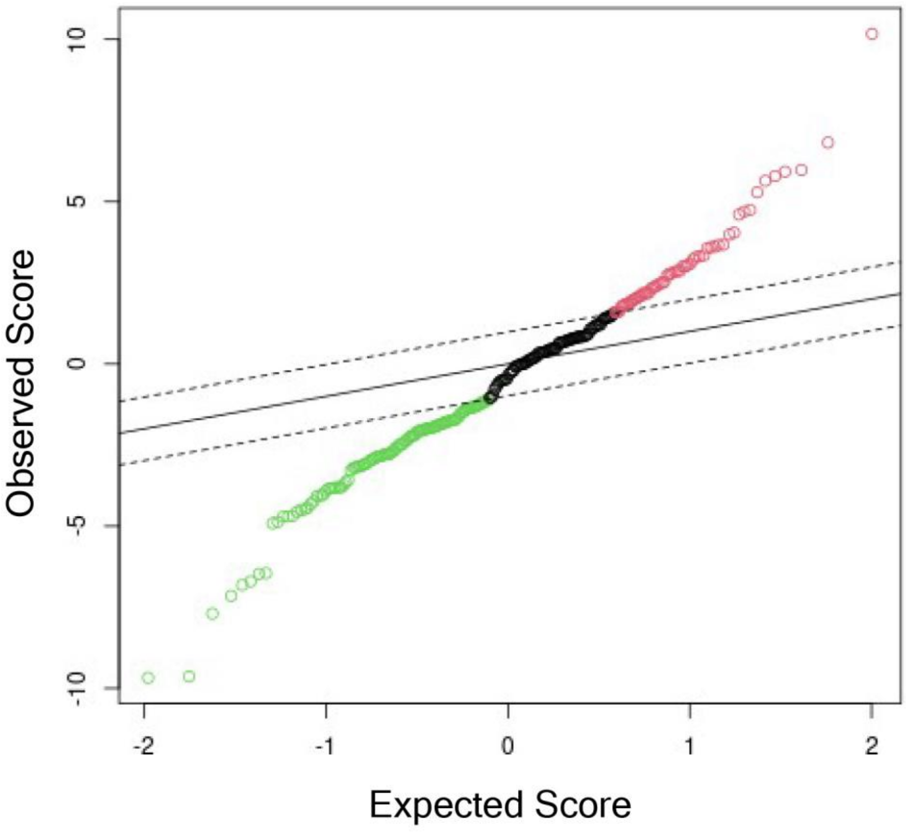

Supplement: Supplementary file 1 — Additional file 1: Figure S1. Statistical Analysis of Microarray. SAM plot of expected vs observed abundance of proteins were plotted. Dashed lines are cut-off for ∆=0.6, corresponding to Q<0.5. Red dots are proteins upregulated by SBP-Ag; green are downregulated. [file 12014_2022_9351_MOESM1_ESM.pdf]
